# Determination of Very Low Concentration of Bisphenol A in Toys and Baby Pacifiers Using Dispersive Liquid–Liquid Microextraction by In Situ Ionic Liquid Formation and High-Performance Liquid Chromatography

**DOI:** 10.3390/ph13100301

**Published:** 2020-10-12

**Authors:** Yesica Vicente-Martínez, Manuel Caravaca, Antonio Soto-Meca

**Affiliations:** Spanish Air Force Academy, University Centre of Defence, Coronel López Peña st., n/n, 30720 Murcia, Spain; yesica.vicente@cud.upct.es (Y.V.-M.); antonio.soto@cud.upct.es (A.S.-M.)

**Keywords:** bisphenol A, high-performance liquid chromatography, ionic liquid, dispersive liquid–liquid microextraction, extraction kinetic studies

## Abstract

Bisphenol A (BPA) is a chemical compound used in the manufacturing of plastics and resins whose presence in the body in low concentrations can cause serious health problems. Due to this, there is a growing interest in the scientific community to develop analytical methods that allow quantifying trace concentrations of BPA in different types of samples. The determination of this compound in toys made of plastics that can be manipulated by children leads to an extra concern, because it is possible for BPA to enter the body by introducing these toys into the mouth. This work presents a novel procedure to the quickly and easily quantification of trace levels of BPA in samples of toys and pacifiers according to the current demanding regulations. The determination of very low levels of BPA was carried out by ionic liquid dispersive liquid–liquid microextraction (IL-DLLME) followed by high-performance liquid chromatography (HPLC). The formation in situ of the ionic liquid (IL) 1-octyl-3-methylimidazolium bis((trifluoromethane)sulfonyl)imide ([C_8_MIm] [NTf_2_]), was achieved by mixing 1-octyl-3-methylimidazolium chloride ([C_8_MIm]Cl) and lithium bis(trifluoromethanesulfonyl)imide ([NTf_2_]Li) aqueous solutions, reaching an instant dispersion whose cloud of microdrops allows the total extraction of BPA in the IL from aqueous solutions. After centrifugation, BPA concentration in the sedimented phase was determined by HPLC. The optimal experimental conditions for the microextraction and determination of BPA in the IL were studied. The total extraction was achieved at pH 4, heating the sample at 30 °C for 5 min, using 100 µL of IL precursor volume, and spinning after the formation of dispersion at 3000 rpm for 10 min. The enrichment factor (EF) and detection limit (LOD) reached with the procedure were 299 and 0.19 µg L^−1^, respectively. The relative standard deviation for ten replications at the 0.5 µg L^−1^ level was 5.2%. Recovery studies showed a mean value for BPA recovery percentage in the samples of 99%. Additionally, a hybrid model was applied to characterize the extraction kinetics. This simple, low cost and fast method simplifies traditional microextraction techniques, representing an outstanding alternative.

## 1. Introduction

Bisphenol A (BPA) is a chemical compound widely used in the manufacturing of plastics and epoxy resins, which are later employed in toys and bottle teats production. Trace residue levels of BPA cause serious problems in the normal function of the endocrine system [1], being a mutagenic and carcinogenic compound. Epidemiologic literature reports relationship between low BPA levels and pubertal development, fetal and childhood growth or metabolic and reproductive diseases, among others [2]. It has been stated that even extremely low doses, parts per trillion, can alter cell function [3]. Consequently, the European Union established a specific migration limit (SML) of 0.04 mg L^−1^ for BPA used in toys for children under 36 months [4]. Moreover, the European Commission established a ban on the use of BPA in the manufacturing of polycarbonate baby bottles for infants in 2011 [5].

The restrictive regulations regarding BPA content in toys and baby bottles makes necessary the development of analytical methods with high sensitivity and low detection limit (LOD) which allows to determinate trace concentrations. This high sensitivity requires preconcentration techniques during the sample preparation step. Microextraction techniques are environmentally friendly methods which have replaced classical methods last years. Nowadays, ionic liquids (IL) are very used as extractant phase to determine trace concentrations of different chemical compounds using liquid–liquid microextraction (LLME) techniques because of their suitable characteristics, such as vapor pressure, solubility, and thermal stability. However, the usual microextraction techniques often require the use of organic solvents acting as dispersing agents, or ultrasound to achieve the total extraction of the compound, also requiring long times and high temperatures for a full extraction [6,7,8,9,10].

Particularly, the determination of BPA has been carried out by many authors from different matrices, such as plastics by means of gas chromatography [11,12], vegetable oils using a magnetic ionic liquid [6], and a variety of foods and drinks by different analytical techniques [13,14,15,16,17,18,19].

In recent years, different chemical sensors have been manufactured for the determination of BPA, based on graphene-palladium nanoparticles/polyvinyl alcohol hybrids [20], using CdO nanoparticles [17], or an amperometric enzyme inhibition biosensor based on xanthine oxidase immobilised onto glassy carbon [21].

However, methods for determining traces of BPA in toys or materials used in baby bottles, such as nipples, have not been practically studied recently, since the sensitivity requirements established by European Regulations are very restrictive [4]. Notwithstanding, several interesting studies can be listed, although they present some limitations. A work carried out in 2012 to determine the content of BPA in toys by gas chromatography-mass spectrometry reached detection limits of 10 µg kg^−1^, being necessary the sample derivatization [16]. A procedure of liquid chromatography with fluorescence detection was developed to determine BPA in toy, the LOD reached equal to 50 µg L^−1^, not realistic in comparison to concentrations found in most toys [22]. Moreover, the concentration of BPA has been determined in toys used by dogs, reaching the quantification of concentrations of µg mL^−1^ [23].

In this work, it is introduced a procedure to simulate the migration of BPA in toys and pacifier teats. These objects were subjected to the conditions of children saliva when sucked, transferring this compound to an aqueous solution [24]. After that, the aqueous solution containing BPA is subjected to a microextraction method based on the in situ formation of IL in a straightforward ion-exchange reaction. An instantaneous dispersion is obtained after mixing the IL precursors, allowing the rapid and total extraction of BPA from aqueous solutions in the IL droplet cloud. In just a few minutes, after centrifuging the dispersion, the sedimented extract at the bottom of the tube is analyzed by HPLC to quantification of amounts of BPA present in the solution. This method allows to determine very low concentrations of BPA in a simple and cost effective-way, in comparison with other techniques requiring more expensive instrumentation. In this way, the procedure is accessible for any standard laboratory.

The experimental conditions to achieve the total extraction of BPA were studied, reached for pH = 4 and 30 °C, in just 5 min. This procedure leads to an enrichment factor (EF) and LOD of 299 and 0.19 µg L^−1^, respectively. These values allow the determination of BPA concentrations under the very restrictive conditions of concentration that the regulations establish. The procedure was validated applying it to determination of BPA in solutions with known concentrations. Recovery, repeatability and reproducibility studies were carried out showing that it is a robust method for the established purpose, becoming a realistic and viable alternative to determine BPA in toys and baby pacifiers.

## 2. Results and Discussion

### 2.1. Optimization of the DLLME Conditions

#### 2.1.1. Optimization of Aqueous Phase and IL Precursors Volume

The aqueous phase volume was studied with different volumes of IL precursors in order to reach the best microextractions conditions. Among them, it is important to get a constant IL sedimented volume after centrifugation, allowing to carry out the measurement by triplicate and to obtain a suitable EF. To this end, aqueous phase volumes of 5, 10, 15 and 20 mL were tested with 25, 50 and 100 µL of each IL precursor. However, regardless of the aqueous phase volume, when the volumes of IL precursors were 25 and 50 µL, the volume of the IL phase sedimented after centrifugation was so small that it was not possible to analyze it.

When a volume of aqueous phase of 5 mL and 100 µL of each IL precursors were employed, the volume of IL formed after centrifugation was 48 µL, resulting in an EF of 104. When the volume of aqueous phase was 10 mL and the volume for each IL precursor was 100 µL, the volume of IL formed after centrifugation was 33 µL, achieving an EF close to 303. A volume equal to 18 µL of IL phase was achieved when the aqueous phase volume was 15 mL and volume of precursor was 100 µL, but not allowing measurements by triplicate. Finally, when the volume of aqueous phase was equal to 20 mL and volumes of precursors were 100 µL each, the volume obtained of IL sedimented was 10 µL, also not allowing measurements by triplicate. Results are summarized in Table 1.

Consequently, a volume of 10 mL of aqueous phase was chosen because for 33 µL of IL the measurements can be carried out by triplicate, still achieving a high EF, close to 300.

#### 2.1.2. Optimization of pH Conditions

**Figure 1:**
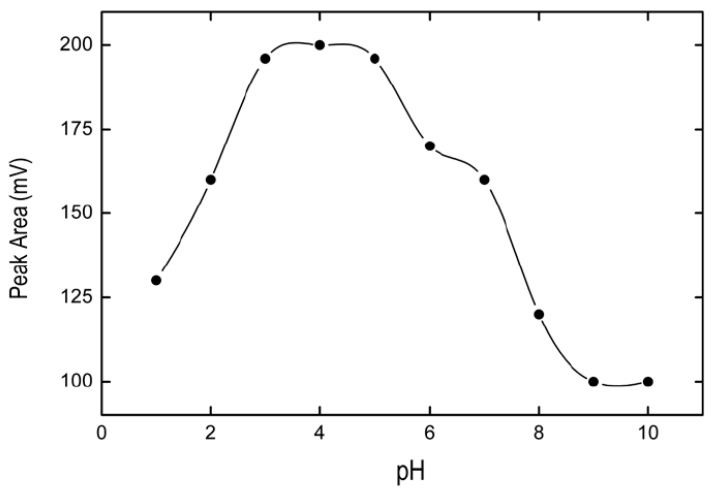
Effect of pH on BPA extraction. Solid line represents spline connectors. As depicted from the plot, a maximum Peak Area was achieved for pH = 4.

In order to determine the adequate pH to reach the maximum efficiency of the extraction of BPA in the IL, several experiments were carried out. A range of pH 1–10 was studied to obtain the best results in the extraction of BPA. Figure 1 shows that the maximum BPA extraction is achieved for a pH range between 3 and 5. For the discussion of this result, this behavior is due to a hydrophobic interaction between IL and BPA which decreases because of the deprotonation of BPA when pH increases [25]. A pH value of 4 was selected as adequate to carry out the extraction.

#### 2.1.3. Optimization of Temperature Conditions and Incubation Time

**Figure 2:**
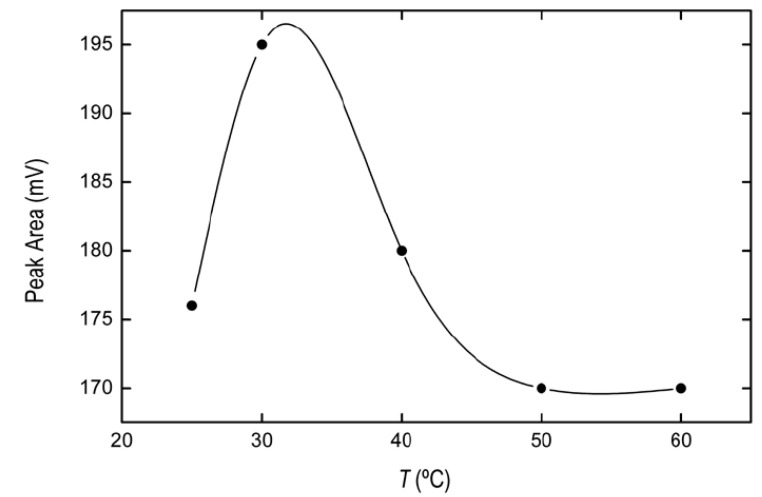
Effect of the temperature on the BPA signal obtained from IL phase. Solid line represents spline connectors. Maximum extraction efficiency was achieved at T = 30 °C.

In the extraction procedure the solution temperature plays a very important role, influencing the extraction efficiency of DLLME. Because of this, the temperature effect was studied within the range 25–60 °C. The aqueous was heated in a thermostatic bath for ten minutes, prior to the addition of the IL precursors at different temperatures belonging to the range mentioned above. As is shown in Figure 2, the best extraction efficiency was obtained at 30 °C. Accordingly, we selected this temperature as the best one for our microextraction process.

**Figure 3:**
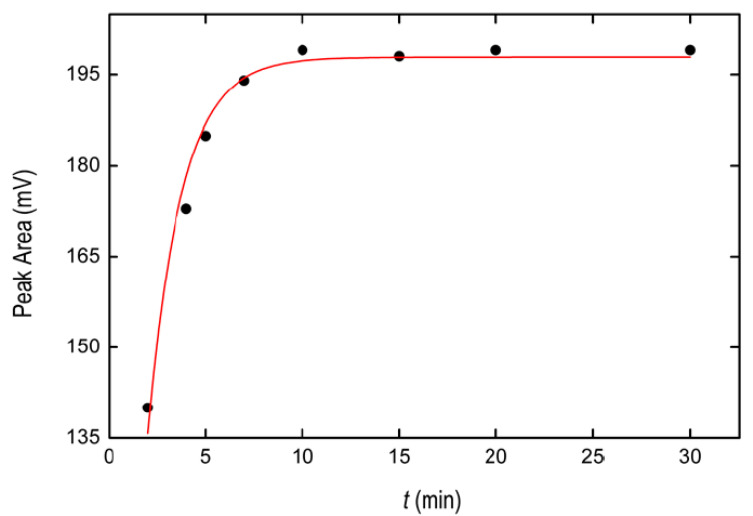
Effect of incubation time at 30 °C on the BPA signal obtained from the IL phase. Solid red line represents the fit to Equation (1).

Additionally, the incubation time of the samples at 30 °C was studied within the range 2–30 min. Figure 3 shows that the extraction efficiency of the BPA in the IL increased from 2 to 10 min, then remaining nearly constant until 30 min. Accordingly, five minutes was selected as the heating time for subsequent measurements. As a discussion, the extraction kinetics was characterized by an efficient hybrid kinetic model, Equation (1) (see Section 3.7), resulting in an adjusted *R*^2^ equal to 0.974. The fit is represented by red solid line in Figure 3.

#### 2.1.4. Optimization of Centrifugation Time

**Figure 4:**
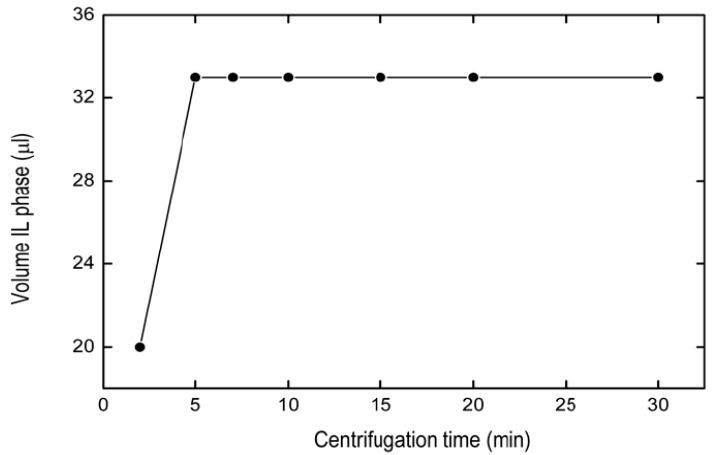
Effect of centrifugation time on volume IL phase. Solid line represents straight connectors.

The centrifugation time was studied in order to obtain the adequate IL volume after the microextraction procedure. Times equal to 2, 5, 7, 10, 15, 20 and 30 min of centrifugation were applied at 3000 rpm. Figure 4 shows that the volume of IL sedimented increased from 0 to 5 min, then remaining constant until 30 min. The choice for the adequate centrifugation time was 10 min.

### 2.2. Analytical Figures of Merit of the Proposed Method

The EF was evaluated from the quotient between the slope obtained in the calibration line, by applying the proposed preconcentration procedure in experimental section, and the slope resulting from directly measuring BPA in aqueous solution. EF achieved a value of 299. This value coincides with the quotient of the volume of the aqueous phase and the volume of the sedimented IL.

The analytes show a linear behavior within the range 0.5–0.3 µg L^−1^ (linear equation: Y = 96.437× + 4.736). The regression coefficient *R*^2^ for the proposed procedure was 0.997.

Ten consecutive experiments were carried out at 0.5 µg L^−1^ level of BPA to estimate the repeatability, being the relative standard deviations (RSDs) 5.2%. The reproducibility was calculated from ten measurements obtained on five consecutive days, obtaining RSDs of 6.5%.

The LOD was calculated using the criterion that the analyte quantity is equivalent to three times the standard error of the calibration slope estimation. This results in a LOD equal to 0.19 µg L^−1^ of BPA. Additionally, the limit of quantification (LOQ) is equal to 0.63 µg L^−1^.

To validate the proposed method, it was applied to aqueous solutions with known concentrations of BPA. Table 2 shows the results obtained. As a discussion, the concentrations obtained after applying the proposed microextraction technique are in agreement, including the standard deviations, to the concentrations of the prepared solutions.

### 2.3. Application of the Procedure to Toys and Baby Pacifier Samples

The common activity of children introducing toys into the mouth causes the possibility that some chemical compounds present in the materials for toy manufacturing are solubilized in the saliva, entering the body and causing health risk in some cases.

Regarding BPA, small doses of this chemical could cause serious health problems [1]. For this reason, the European Union established the maximum specific migration limit of BPA in baby toys at 0.04 mg L^−1^ [4]. The pacifier limit has not been established, assuming that they must contain 0% BPA in their composition. Since the elimination mechanisms in babies are not fully operating before 6 months, any exposure to this substance is not considered as safe.

Although the limit of migration of BPA in toys is established in the aforementioned concentration, it is necessary to have analytical methods that allow a safe quantification of much lower levels in materials used by babies, since it constitutes a real concerning danger.

The analytical procedure proposed in this work was applied to determination of BPA in toys and baby pacifiers. For this, it is necessary to submit these materials to a procedure that simulates the migration of chemical in the baby’s saliva. The treatment is regulated by the European Union, consisting of putting 10 cm^2^ of the material in continuous agitation with 100 mL of water for one hour at room temperature [4]. The aqueous solutions were later subjected to the proposed microextraction procedure by in situ formation of an IL. Regarding the toys employed in our study, we chose the samples randomly, among standard small toys from local toy stores in Murcia, Spain. Date of purchase was November 2019 for samples 1–8, and September 2020 for samples 9–11. The values found by application of the procedure under study to several toys are shown in Table 3. All values lie well below the permitted limit. Additionally, recovery studies were carried out for every sample. As can be seen in Table 3, recoveries ranged from 97% to 102%, thus confirming the reliability of the procedure.

To discuss the results, it is worth pointing out that trace BPA levels as those shown in Table 3 could be dangerous, even fulfilling the restrictions of the EU, as reported in the literature [2,3]. Toys and pacifiers add to other BPA sources, being food intake the primary route of human exposure [26,27]. The environment is generally considered as a secondary source and, in particular, it has been stated that it is unlikely to inhale high BPA levels from air, except maybe with the workers of BPA-based products companies [27]. The European Food Safety Authority established in 2015 [28] an average BPA dietary intake reaching 0.857 µg/kg BW/day for infants, and a non-dietary of 0.015 µg/kg BW/day. However, recent studies show that toy BPA intake can approach 246 ng/kg BW/day [29]. The EU set in 2017 a temporary tolerable daily intake (TDI) equal to 4 µg/kg BW/day, of which 10% allocates to exposure to BPA from toys [4]. Additionally, it has been reported than lower levels, such as 0.025–0.2 µg/kg BW/day can lead to severe health problems [27], so again it is emphasized that efficient detection of trace levels could be of paramount importance.

## 3. Materials and Methods

### 3.1. Chemicals and Materials

The bisphenol A standard was purchased from Sigma-Aldrich Chemie (Schnelldorf, Germany). Pure water was prepared using a water purification system formed by a delimer (CILIT MINICRONO (Cilit, SA, Barcelona, Spain) and a water purification system SETA OSMO BL-6 (Sociedad Española de Tratamiento de Agua, Madrid, Spain). Ultrapure water was employed for the preparation of 0.1 M solutions of these ILs.

Bis(trifluoromethane)sulfonamide lithium salt and 1-butyl-3-methylimidazolium chloride (Sigma-Aldrich Chemie, Schnelldorf, Germany) were used in orden to form the IL. Analytical grade acetonitrile was obtained from Applichem Panreac (Darmstadt, Germany), and water for HPLC from Macron Fine Chemicals (Gliwice, Poland). The non-ionic surfactant Triton X-114 was purchased from Sigma-Aldrich (Raleigh, NC, USA). Micro pipettes of 100–1000 µL were brought from Thermo Scientific (Wantaa, Finland).

### 3.2. General Procedure

Sample solution of 10 mL containing BPA in the range of 0.5–3 µg L^−1^ was heated at 30 °C for 5 min. Subsequently, 20 µL of a regulatory dissolution of pH = 4, 100 µL of the [C_8_MIm]Cl solution, 100 µL of 0.1 mol L^−1^ Triton X-114 solution and 100 µL of the [NTf_2_]Li were added. A turbid dispersion was instantaneously formed because of the IL formation reaction from its precursors. The mixture was centrifugated at 3000 rpm for 10 min. The IL sedimented at the bottom of the tube was collected, being 10 µL injected in HPLC using a chromatographic-type syringe. BPA concentrations were determined using the conditions described throughout the manuscript. The measurements were carried out by triplicate.

### 3.3. Procedure Applied to Plastic Toys and Baby Pacifiers

Eleven toy and baby pacifier samples were acquired in local stores and pharmacies, respectively in Murcia, Spain. The toy samples included one plastic blue ball (sample 1), one plastic red ball (sample 2), one plastic blue ball (sample 9), a yellow horse (sample 10), an orange giraffe (sample 11) and three different plastic fruits: one yellow banana (sample 6), one green apple (sample 7) and one purple grape (sample 8). Regarding the baby pacifiers, two samples manufactured after the modification of legislation in specific limit values for chemicals used in toys (samples 3 and 4), and one sample manufactured before legislation of BPA was enforced (sample 5).

A surface of 10 cm^2^ of material from each toy and baby pacifier were contacted under mechanical agitation with 100 mL of water for 1 h with magnetic stirring at room temperature [4]. Then, 10 mL from each aqueous solution were treated with the general procedure.

### 3.4. Ionic Liquid as Acceptor Phase

Organic solvents as a BPA extracting medium have been extensively studied and used over the years [30,31,32]. In recent years, liquid–liquid microextraction techniques have been widely used to extract BPA using different organic solvents, such as benzene [33], hexane [34], ethyl acetate [35] or ethanol [36]. However, the use of IL as an extracting medium instead of the commonly used organic solvents provides a series of advantages, such as low solubility in water and low viscosity, a double condition which is not easily achieved [9,10]. Moreover, mixing water-soluble IL precursors leads to an in situ metathesis reaction, immediately giving rise to a water-immiscible IL [8]. This IL formation reaction provides a dispersed phase through a cloud of microdroplets that allows to get a large surface in contact, thus proportioning the right medium for instantaneous extraction of the analyte.

Excellent results were found when 1-octyl-3-methylimidazolium chloride ([C_8_MIm]Cl) and bis(trifluoromethanesulfonyl)imide ([NTf_2_]Li) were employed as IL precursors. Both precursors were mixed and a turbidity due to the formation of insoluble 1-octyl-3-methylimidazolium bis((trifluoromethane)sulfonyl)imide ([C_8_MIm] [NTf_2_]) was immediately observed by the metathesis reaction:

$$[C_{8}\mathrm{MIm}]\mathrm{Cl}+[{\mathrm{NTf}}_{2}]\mathrm{Li}\to [C_{8}\mathrm{MIm}][{\mathrm{NTf}}_{2}]+\mathrm{LiCl}$$

This procedure greatly simplifies traditional microextraction techniques because it avoids the use of dispersing agents, organic solvents or mechanical techniques, such as ultrasound. Moreover, this method reduces the time necessary to achieve full extraction of the analytes. It has been used for the extraction of several metals and other aromatic species [8,9,10].

Different volumes of [C_8_MIm]Cl and [NTf_2_]Li were studied in order to obtain the best conditions in terms of formed IL volume and reproducibility of the process. The best results were found employing 100 µL of 1 mol L^−1^ [C_8_MIm]Cl and 100 µL of 1 mol L^−1^ [NTf_2_]Li, and a aqueous phase of 10 mL. After centrifugation, 33 µL of IL [C_8_MIm][NTf_2_] were recovered in the bottom of the tube. This volume is in agreement with the theoretical predictions, taking into account the [C_8_MIm][NTf_2_] density [37].

### 3.5. Non-Stick Agent Selection

In order to avoid that IL remains in the walls of the tube during microextraction process, thus collecting the maximum volume of IL, some authors have suggested the employment of surfactants as non-stick agents [9,10,38]. Different volumes and concentrations of surfactants were studied, achieving the best results with the use of Triton X-114. By using 200 µL of aqueous solution 0.2 mol L^−1^ of Triton X-114 at 10 mL of donor phase, the volume of sedimented IL reached a maximum, constant and reproducible value of 33 µL under the experimental conditions proposed, thereby preventing it from sticking to the walls of the container.

### 3.6. Instrumentation and Analytical Conditions

The measurements made in this work were carried out on an HPLC JASCO BS-4000 system (Madrid, Spain), equipped with a sample injector and an ultraviolet detector (UV-4075) operating at 230 nm.

The column used was C18 column (150 × 4.6 mm i.d., 5 µm), and the mobile phase employed consisted in a mixture of 60% acetonitrile and 40% water, at a flow rate of 0.8 mL min^−1^ and an injection volume of 10 µL.

An ultrasonic bath with a HD-5L heating system (P-Selecta S.A., Barcelona, Spain) at 40 kHz of frequency and 60W of power was used for raising the temperature up to 30 °C. An EBA 8 centrifuge (P-Selecta S.A., Barcelona, Spain) was used to disrupt sample emulsions.

### 3.7. Microextraction Kinetic Studies

Characterization of BPA microextraction kinetics was performed through a nonlinear fit of the experimental data, specifically the dependence of Peak Area (mV) on exposure time (min), described by the following hybrid model [39]:

(1) $$\mathrm{Peak}\;\mathrm{Area}=\left(\alpha -\beta \right)\frac{\left(\beta /\alpha \right)e^{\left(\beta -\alpha \right)\gamma t}}{\left(\beta /\alpha \right)e^{\left(\beta -\alpha \right)\gamma t}-1}+\beta$$

where and *α*, *β*, *γ* represent characteristic parameters and *t* is the exposure time.

## 4. Conclusions

Toys and baby pacifiers are subjected to high restrictions of BPA concentrations. This fact makes it difficult in the development of analytical methods capable of quantifying very low levels of this chemical in those materials. However, very low levels of this BPA can seriously affect children’s health. This work presents a novel method of determining very low concentrations of BPA in toys and baby pacifiers as an alternative to the traditional methods for its analysis.

The procedure consists of the in situ formation of an ionic liquid through a metathesis reaction of its precursors, providing a dispersed medium which allows the instantaneous extraction of BPA in IL under very mild and simple experimental conditions. After centrifuging the mixture, the IL sediments at the bottom of the tube and the BPA can be quantified by direct injection of the IL on HPLC. A high enrichment factor (EF = 299) and a low detection limit (LOD = 0.19 µg L^−1^) were achieved. Furthermore, the proposed method presents a high reproducibility and repeatability. It was validated using aqueous solutions of known concentrations of BPA and was successfully applied to real samples of toys and pacifiers complying with the current regulations for it.

## Figures and Tables

**Table 1:** IL volume and EF obtained after centrifugation for different aqueous phase volumes and a fixed IL precursors volume of 100 μL.

Aqueous Phase Volume (mL)	IL Precursors Volume (μL)	IL Volume after Centrifugation (μL)	EF
5	100	48	104
10	100	33	303
15 *	100	18	833
20 *	100	10	2000

* Measurements not achieved by triplicate.

**Table 2:** Results of validation of the proposed procedure in aqueous solutions presenting known concentrations of BPA.

Sample of Known Concentration	Concentration (µg L^−1^)	Content Found ^a^ (µg L^−1^)
blank	0.0	0.01 ± 0.00
1	0.30	0.303 ± 0.002
2	0.40	0.401 ± 0.001
3	0.50	0.500 ± 0.003

^a^ Mean value ± standard deviation (*n* = 3).

**Table 3:** BPA determined from toy and pacifier samples.

Sample	Content Found ^a^ (µg L^−1^)	Recovery %
1—Blue ball	0.30 ± 0.02	100
2—Red ball	ND	101
3—White teat	0.23 ± 0.01	97
4—Pink teat	ND	-
5—Red teat	ND	-
6—Yellow banana	0.25 ± 0.01	102
7—Green apple	0.22 ± 0.04	101
8—Violet grape	0.30 ± 0.03	99
9—Blue ball	0.28 ± 0.02	98
10—Yellow horse	0.21 ± 0.02	101
11—Orange giraffe	0.25 ± 0.04	98

ND: Not detected; ^a^ Mean value ± standard deviation (*n* = 3).
